# Attenuation of Palmitic Acid-Induced Intestinal Epithelial Barrier Dysfunction by 6-Shogaol in Caco-2 Cells: The Role of MiR-216a-5p/TLR4/NF-κB Axis

**DOI:** 10.3390/metabo12111028

**Published:** 2022-10-26

**Authors:** Fangxin Ouyang, Bo Li, Yuli Wang, Longhua Xu, Dapeng Li, Feng Li, Dongxiao Sun-Waterhouse

**Affiliations:** 1 College of Food Science and Engineering, Shandong Agricultural University, 61 Daizong Street, Taian 271018, China; 2 Department of Nursing, Jinan Vocational College of Nursing, 3636 Gangxi Road, Jinan 250102, China; 3 School of Chemical Sciences, The University of Auckland, Private Bag 92019, Auckland 1142, New Zealand

**Keywords:** intestinal epithelial barrier, 6-shogaol, palmitic acid, tight junctions, miR-216a-5p/TLR4

## Abstract

Palmitic acid (PA) can lead to intestinal epithelial barrier dysfunction. In this study, the protective effects and working mechanisms of 6-shogaol against PA-induced intestinal barrier dysfunction were investigated in human intestinal epithelial Caco-2 cells. Transepithelial electrical resistance (TEER), paracellular flux, qRT-PCR, immunofluorescence, and Western blot experiments showed that the 24-h treatment with 400 μM PA damaged intestinal barrier integrity, as evidenced by a reduction of 48% in the TEER value, a 4.1-fold increase in the flux of fluorescein isothiocyanate-dextran 4000 (FD-4), and decreases in the mRNA and protein expression of tight junction (TJ)-associated proteins (claudin-1, occludin, and ZO-1), compared with the control. The PA treatment significantly (*p* < 0.05) increased the levels of pro-inflammatory cytokines (interleukin (IL)-6, IL-1β, and tumor necrosis factor-alpha (TNF-α)) in Caco-2 cells due to the upregulation of toll-like receptor 4 (TLR4), myeloid differentiation factor 88 (MyD88), phosphorylated nuclear factor kappa-B (NF-κB) proteins, and downregulation of miR-216a-5p (which directly targeted TLR4). Co-treatment with PA and 6-shogaol (2.5 μM) significantly (*p* < 0.05) attenuated PA-induced changes through regulation of TJs via the miR-216a-5p/TLR4/NF-κB signaling pathway. This study provides insights into the functions and working mechanisms of 6-shogaol as a promising food-derived agent against PA-induced intestinal epithelial barrier dysfunction.

## 1. Introduction

Intestinal epithelial tight junctions (TJs) are located at the boundary between the apical and lateral membrane surfaces of the neighboring epithelial cells. TJs have over 50 types of proteins, including transmembrane and intracellular scaffold proteins such as claudins, occludin, cytoplasmic scaffolding ZO proteins, and junctional adhesion molecules (JAMs) [1]. Together with the intestinal epithelial cells, TJs form a selective and permeable physical barrier between epithelial and endothelial cells to allow directional absorption of ions, nutrients, and water while resisting/excluding detrimental luminal contents such as intestinal pathogens, antigens, and toxins [2]. The intestinal barrier is highly sensitive to stressors, diet, and microbiota changes. Intestinal barrier dysfunction leads to increased invasion of harmful substances, including intestinal pathogenic bacteria or toxins, causing local and systemic inflammatory reactions and eventually a variety of diseases such as bacterial intestinal infections, inflammatory bowel disease (IBD), irritable bowel syndrome (IBS), and food allergies [3]. Therefore, the integrity and function of the intestinal barrier, including TJs, are critical for gut homeostasis. Correcting intestinal barrier dysfunction represents a therapeutic approach for treating the above-mentioned diseases.

A widespread shift from traditional diets towards prevalent Western diets high in fats, sugars, and highly processed foods in modern society, along with a rise in sedentary lifestyles, causes significant distortions of human metabolism and harmful effects on human health. Multiple lines of evidence reveal that high-fat and/or high-sugar diets have adverse effects on the human body, including the promotion of gastrointestinal dysfunction and disorders, through decreasing microbial diversity, disrupting membrane integrity, and triggering local and systemic inflammations [4,5]. Palmitic acid (PA) is one of the most abundant saturated fatty acids in human diets and has almost the greatest presence in the bloodstream fat tissues and plasma lipoproteins among the fatty acids [6]. It was found that high-fat diets (HFDs) led to a significant increase in blood’s PA level, which was closely associated with intestinal barrier dysfunction [7]. Among the diets rich in different types of oils, the PA-based diet led to the most active transport of lipopolysaccharide (LPS) to the body tissues and the highest levels of plasma interleukin 6 (IL-6) in mice [8]. PA can induce intestinal dysfunctions by targeting barrier integrity and inflammation, i.e., impairing paracellular permeability and TJs’ expression and localization while stimulating the production of (pro-)inflammatory cytokines in cells [9,10]. A recent in vivo study revealed that the mice administrated PA by gavage had higher intestinal permeability, less TJ expression and mislocalization, and higher IL-1β levels compared with the control group [10]. These findings suggest that excessive consumption of PA induces intestinal barrier dysfunction by disrupting the integrity of TJs and triggering inflammation response.

6-Shogaol is a major active compound in dried ginger rhizomes, and has received increasing attention due to its antioxidant, anti-inflammatory, and anticarcinogenic activities [11]. Compared with the most active compound in fresh ginger, 6-gingerol, 6-shogaol possesses higher biological activity and stability [12]. Previously, we found that compared with other gingerols, 6-shogaol had greater potential in suppressing the LPS-induced production of nitric oxide (NO) in mouse macrophages RAW264.7 [13]. In a mouse model of Parkinson’s disease (PD), 6-shogaol counteracted the decreases of occludin and ZO-1 proteins and reduced the production of tumor necrosis factor-alpha (TNF-α) and IL-1β in the colon, thereby preventing inflammation-induced gastrointestinal dysfunction [14]. In the human intestinal cell models, HT-29/B6 and Caco-2, 6-shogaol exhibited protective effects against TNF-α-induced barrier loss by attenuating the TNF-α-initiated reduction of transepithelial electrical resistance (TEER) and increases in luciferin permeability and protein expression of the claudin-1 protein [15]. Accordingly, 6-shogaol may be a promising protective agent against intestinal barrier dysfunction.

MicroRNAs (miRNAs) are a subclass of small non-coding RNAs that participate in a variety of biological processes, in particular, RNA silencing, post-transcriptional and -translational regulation, and intercellular communication mediation. Recent studies have highlighted the important regulatory roles of miRNAs in relation to various diseases, including intestinal barrier dysfunction. MiR-24 was found to participate in the regulation of the intestinal barrier and inflammation in ulcerative colitis patients [16]. Recently, miR-216a-5p was found to be able to alleviate LPS-induced vascular endothelial injury by targeting toll-like receptor 4 (TLR4) [17]. MiR-216a-5p may also inhibit microglia-induced neuroinflammation by targeting the TLR4 signaling pathway [18]. The activation of TLR4 is known to trigger its downstream effector, nuclear factor kappa-B (NF-κB), which is then transported into the nucleus to enhance the expression of pro-inflammatory cytokines [19]. Accordingly, it is of interest whether 6-shogaol has a protective effect against PA-induced intestinal barrier dysfunction and such a process involves miR-216a-5p, TLR4, and NF-κB. This study was set up to fill this knowledge gap. PA-treated human epithelial colorectal adenocarcinoma (Caco-2) cells were used as the intestinal barrier dysfunction model to examine the protective effect of 6-shogaol.

## 2. Materials and Methods

### 2.1. Chemicals and Reagents

6-Shogaol (≥98%) was purchased from Shanghai Yuanye Company (Shanghai, China). Palmitic acid (PA; purity ≥ 98%) and fluorescein isothiocyanate-dextran 4000 (FD-4; molecular weight, 4 kDa) were purchased from Sigma-Aldrich (St. Louis, MO, USA). Dimethyl sulfoxide (DMSO), penicillin-streptomycin, 3-(4,5-dimethylthiazol-2-yl)-2,5-diphenyl tetrazolium bromide (MTT), and 0.25% trypsin-EDTA were purchased from Solarbio Life Sciences Co. (Beijing, China). Dulbecco’s Modified Eagle’s minimum essential medium (DMEM) and fetal bovine serum (FBS) were purchased from Gibco (Rockville, MD, USA). Enzyme-linked immunosorbent assay (ELISA) kits for the determination of TNF-α, IL-1β, and IL-6 were obtained from Lengton Inc. (Shanghai, China). All other chemicals and reagents were of analytical grade and obtained from Sinopharm Chemicals Co. Ltd. (Shanghai, China).

### 2.2. Cell Culture and Treatments

Caco-2 cells were obtained from American Type Culture Collection (ATCC, Rockville, MD, USA), and maintained at 37 °C and under a 5% CO_2_ atmosphere in DMEM supplemented with 10% FBS, 100 U/mL penicillin, and 100 μg/mL streptomycin mixture. Cells at passages 4 to 12 were used for all the experiments. The differentiated Caco-2 cells refer to those monolayers of mature intestinal cells that were subjected to 21-day culture and exhibited enterocyte-like characteristics. PA and 6-shogaol were dissolved, respectively, in DMSO to obtain stock solutions, which were further diluted to the desired concentrations with the basic medium before use. The cells were subjected to three different treatments for 24 h: PA (400 μM), 6-shogaol (2.5 μM), and PA (400 μM) + 6-shogaol (2.5 μM). The cells cultured solely in the basic medium were used as the control group. For the permeability experiments, the cells (density: 1 × 10^5^ cells/mL) were inoculated into the chambers of the transwell inserts (6.5 mm, 0.4 μm pore polycarbonate membrane) in 24-well plates (Corning, NY, USA), and incubated at 37 °C and under a 5% CO_2_ atmosphere for 21 days. For the immunofluorescence, ELISA, Western blot, and quantitative real-time PCR (qRT-PCR) experiments, cells (density: 2 × 10^5^ /well) were cultured in 6-well plates.

### 2.3. Cell Viability Evaluated by the MTT Assay

The influences of 6-shogaol and/or PA on cell viability were evaluated by the MTT assay. Caco-2 cells (density: 4 × 10^4^ cells/mL) were incubated in 96-well plates for 24 h. The medium was then replaced with serum-free basic medium containing a diluted 6-shogaol solution (0.625–40 μM) or PA solution (100–800 μM), before the cells were incubated for another 24 h. At the end of the incubation, the medium was discarded and an aliquot (100 μL) of MTT dye solution (0.5 mg/mL in PBS) was added to each well. The plates containing cells and dye were incubated at 37 °C and under a 5% CO_2_ atmosphere for 4 h. After the addition of DMSO to each well, the absorbance at 570 nm was measured by a Multiskan MK3 microplate reader (Thermo Fisher Scientific, Waltham, MA, USA). The relative cell viability was expressed as a percentage (%) of proliferation relative to the untreated control cells.

### 2.4. Determination of Cell Permeability

The epithelial barrier function was assessed based on the transepithelial electrical resistance (TEER) and paracellular permeability. TEER in inverse proportion to the permeability of small ions was determined according to a previous procedure [10]. Briefly, Caco-2 cells (100 μL; density: 1 × 10^5^ cells/mL) were inoculated into the transwell inserts of the 24-well plates (Corning, NY, USA), before the addition of 600 μL of culture medium to the basolateral compartment. The medium was replaced with fresh culture medium every other day in the first 7 days then every day. The TEER value was monitored on day 4, 7, 11, 15, 19, and 21 using a Millicell-ERS-2 Volt-Ohm meter (Millipore, Burlington, MA, USA).

To assess the paracellular permeability, an aliquot (100 μL) of 1 mg/mL FD-4 was added to the AP side while an aliquot (600 μL) of D-Hanks buffer solution (GENOM, Hangzhou, China) was added to the BL side. After the cells were incubated for 2 h, an aliquot (200 μL) of subsample was withdrawn from the BL side, which was quantified fluorometrically using a multi-mode microplate reader (SpectraMax M5; Molecular Devices, San Jose, CA, USA) set at an excitation wavelength of 480 nm and emission wavelength of 520 nm. The apparent permeability coefficient (P_app_) was calculated according to a previous method [20].

### 2.5. Total RNA Extraction and qRT-PCR

Total RNA was extracted with the FastPure Cell/Tissue Total RNA Isolation Kit V2 from Vazyme (Nanjing, China) according to the manufacturer’s protocol. Reverse transcription was performed using an Evo M-MLV RT Mix Kit with the gDNA Clean for qPCR AG11728 or miRNA 1st strand cDNA synthesis kit AG11717 (Accurate Biotechnology, Hunan, China). qRT-PCR was performed using an ABI 7500 Fast Real-Time PCR system (Applied Biosystems, MA, USA) with an SYBR Premix Kit AG11701 (for mRNA) or AG11702 (for miRNA) (Accurate Biotechnology, Hunan, China). The levels of gene expression were quantified by the 2^−ΔΔCt^ method. GAPDH was used as the reference gene for mRNA, and U6 was used as the control for miRNA. The primer sequences used are listed in Table 1, and they were purchased from Sangon Biotech (Shanghai, China).

### 2.6. Western Blot Analysis

The cells were lysed for 20 min in 180 μL of lysis buffer (Beyotime, Shanghai, China) on ice before the centrifugation at 4 °C and 14,000 rpm for 10 min. The total protein concentration was estimated using a bicinchoninic acid (BCA) protein detection kit (CWBIO, Shanghai, China) with bovine serum albumin (BSA) as the standard. The proteins were separated by sodium dodecyl sulfate poly-acrylamide gel electrophoresis (SDS-PAGE) and transferred onto a polyvinylidene fluoride (PVDF) membrane (Millipore, Bedford, MA, USA). After being blocked with 5% (*w/v*) skimmed milk for 2 h, the membranes were probed with corresponding primary antibodies overnight at 4 °C and then incubated with secondary antibodies conjugated with horseradish peroxidase for 2 h. The primary antibodies used were: TLR4 (1:1000, Abcam, ab13556), myeloid differentiation factor 88 (MyD88, 1:1000, Abcam, ab133739), nuclear factor kappa-B (NF-κB) p65 (1:1000, Abcam, ab32536), NF-κB p65 (phospho S536) (1:1000, Abcam, ab76302), claudin-1 (1:2000, Abcam, ab211737), occludin (1:1000, Abcam, ab216327), ZO-1 (1:1000, Abcam, ab276131), myosin light-chain kinase (MLCK, 1:1000, ABclonal, A3835), and β-actin (1:5000, Abcam, ab179467). The protein intensity was quantified using the Image J software (version 1.8.0, NIH, Bethesda, MD, USA).

### 2.7. Immunofluorescence Analysis

After the treatment as described in Section 2.2., the cells were fixed in freshly prepared 4% paraformaldehyde-neutral PBS at room temperature for 20 min, and treated with 0.5% TritonX-100 for 20 min and then 5% goat serum for 1 h. The resulting cells were probed at 4 °C overnight with a corresponding primary antibody (i.e., claudin-1 (1: 1000, Abcam, ab211737), occludin (1:100, Abcam, ab216327), or ZO-1 (1:100, Abcam, ab221547)). Then, the cells were washed three times with PBS and incubated with the Alexa Fluor^®^ 488 fluorescent secondary antibody (1:300, Abcam, AB150077) for another hour. The fluorescence of at least six regions for the sample of each group was examined under an inverted BX-FM fluorescence microscope (Olympus, Tokyo, Japan). The cell nuclei were stained with 4′,6-diamidino-2-phenylindole (DAPI) (Beyotime, Shanghai, China). Image J software was used to determine the integrated density.

### 2.8. Determination of Pro-Inflammatory Cytokines by ELISA

Caco-2 cells were lysed on ice for 20 min with a lysis buffer containing phenylmethane sulfonyl fluoride (PMSF), before centrifugation at 14,000 rpm and 4 °C for 10 min. The resulting supernatant was collected for the quantification of TNF-α, IL-1β, and IL-6 using commercial ELISA kits.

### 2.9. Bioinformatics Analysis

The target gene prediction was performed using the following databases: TargetScan (http://www.targetscan.org/, accessed on 10 September 2021), miRDB (http://mirdb.org/, accessed on 11 September 2021), and PicTar (http://pictar.mdc-berlin.de/, accessed on 11 September 2021).

### 2.10. MiR-216a-5p Transfection

Caco-2 cells (density: 1 × 10^5^/mL) were seeded in 6-well plates and transfected using Micropoly-transfecter^TM^ cell reagents (Micropoly Biotech Co., Nanjing, China) according to the manufacturer’s instructions. MiR-216a-5p mimics, negative control (NC), miR-216a-5p inhibitor, and microRNA inhibitor NC were synthesized by GenePharma (Shanghai, China). The transfection efficiency was evaluated by qRT-PCR after each transfection.

### 2.11. Statistical Analysis

All experiments were carried out in triplicate, and the data are expressed as means ± standard deviations. A one-way analysis of variance (ANOVA), along with Duncan’s multiple range test, was performed to estimate the significant differences between the data using the SPSS 23.0 statistical software package (IBM Corp., Armonk, NY, USA). A *p* value lower than 0.05 indicates that the difference was statistically significant.

## 3. Results

### 3.1. Cytotoxicity of 6-Shogaol and PA

**Figure 1:**
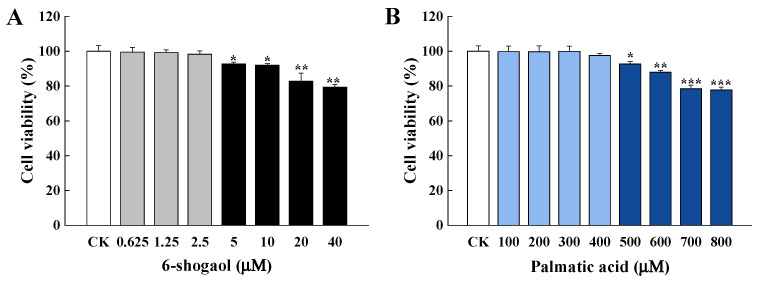
Effects of 6-shogaol and palmitic acid (PA) on the cell viability of Caco-2 cells: (**A**) Relative cell viability of Caco-2 cells treated with 6-shogaol (0.625, 1.25, 2.5, 5, 10, 20, or 40 μM) for 24 h; (**B**) Relative cell viability of Caco-2 cells treated with PA (100, 200, 300, 400, 500, 600, 700, or 800 μM) for 24 h. Data are given as the mean ± standard deviation (n = 3). * *p* < 0.05, ** *p* < 0.01, *** *p* < 0.001 vs. the control.

The cytotoxicity of 6-shogaol or PA was evaluated based on the cell viability of Caco-2 cells incubated for 24 h with 6-shogaol (0.625~40 μM) or PA (100~800 μM). As shown in Figure 1A, there were no significant differences in the cell viability between the control group and those treated with 6-shogaol < 5 μM. However, the relative viability significantly (*p* < 0.05) decreased when 6-shogaol was 5 μM or higher. Figure 1B shows that PA at higher concentrations (500~800 μM) significantly (*p* < 0.05) decreased the cell viability compared with the control, but PA in the range of 100~400 μM had no effect on the cell viability. Accordingly, PA at 400 μM was selected for establishing the intestinal barrier injury model whilst 6-shogaol at 0.625, 1.25, or 2.5 μM was used for subsequent intervention experiments.

### 3.2. 6-Shogaol Alleviated PA-Induced Intestinal Barrier Dysfunction in Caco-2 Cells

**Figure 2:**
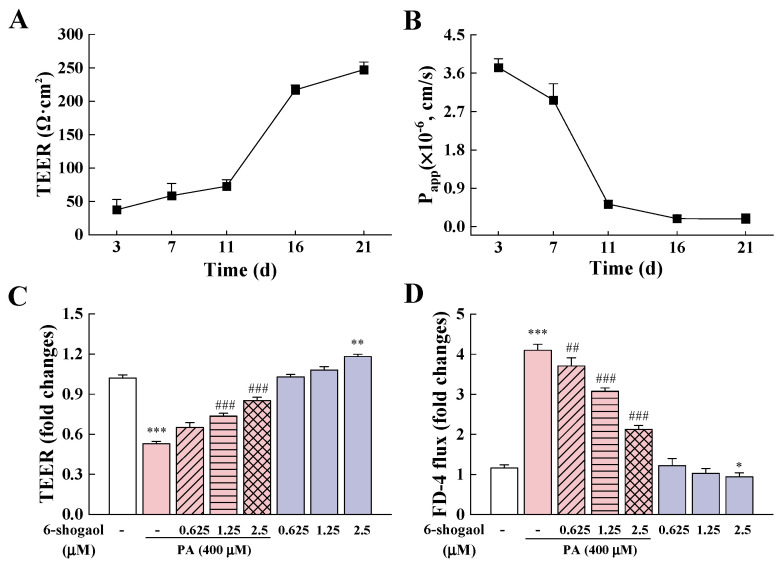
6-Shogaol alleviated PA-induced intestinal epithelial barrier dysfunction in Caco-2 cells: (**A**) Changes in transepithelial electrical resistance (TEER) during Caco-2 cell modeling; (**B**) FD-4 flux changes during Caco-2 cell modeling; (**C**) Effects of 6-shogaol on TEER in PA-treated Caco-2 monolayers; (**D**) Effects of 6-shogaol on the permeability of PA-treated Caco-2 monolayers. Data are given as means ± standard deviations (n = 3). * *p* < 0.05, ** *p* < 0.01, *** *p* < 0.001 vs. the control; ^##^ *p* < 0.01, ^###^ *p* < 0.001 vs. the PA-treated group.

To examine whether 6-shogaol exerts protective effects on Caco-2 cells against PA-induced intestinal epithelial barrier disruption, TEER and paracellular flux assays were performed. After the 21-day differentiation, the cells showed a TEER value of 247.17 ± 11.37 Ω·cm^2^ and *p*_app_ value of 0.18 × 10^−6^ cm/s (Figure 2A,B). Previous studies showed that 200 Ω·cm^2^ might be the basic value for cell monolayer formation [21]. The results obtained in this research indicate the success of constructing mature Caco-2 monolayers with developed enterocyte characteristics. Compared with the control, the 24-h PA treatment led to a significant reduction (48%) in the TEER value (Figure 2C) and a 4.1-fold increase in the FD-4 flux (Figure 2D), indicating that the PA treatment disrupted the integrity of the Caco-2 epithelial barrier. Notably, 6-shogaol significantly (*p* < 0.05) increased the TEER values of Caco-2 cells in a dose-dependent manner but decreased the FD-4 flux compared with the PA alone treatment. The cells treated with 2.5 μM 6-shogaol had a significantly (*p* < 0.05) higher TEER value and a lower FD-4 flux than the control group, indicating that 6-shogaol could improve the intestinal barrier function of Caco-2 cells in the absence of PA.

### 3.3. 6-Shogaol Protected Caco-2 Cells against PA-Induced TJ Damage

**Figure 3:**
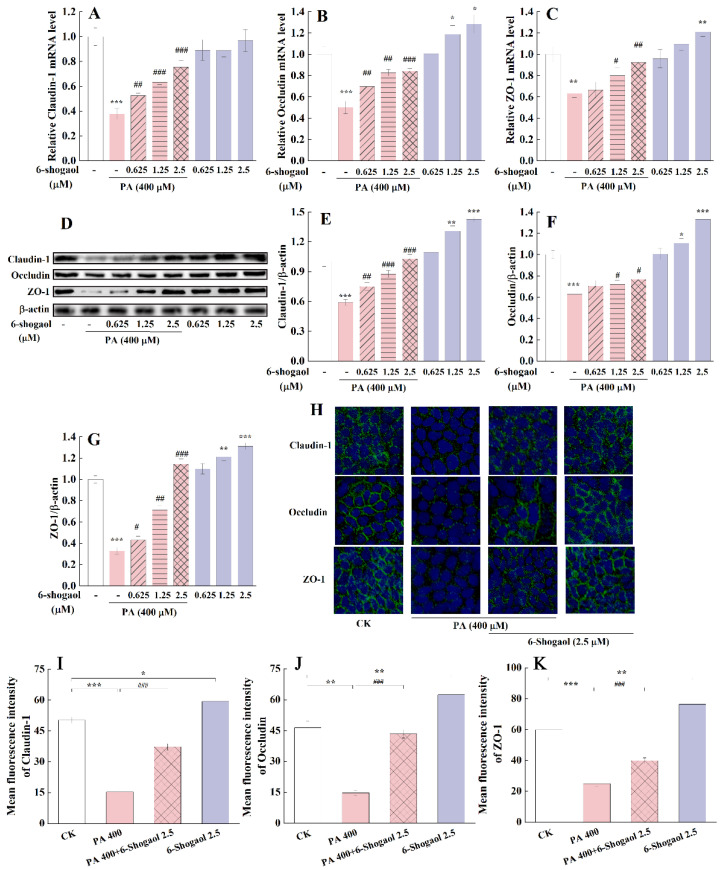
6-Shogaol attenuated PA-induced tight junction (TJ) dysfunction in Caco-2 cells: (**A**) Claudin-1 mRNA; (**B**) Occludin mRNA; (**C**) Zonula Occluden-1 (ZO-1) mRNA; (**D**) Representative immunoblotting of TJ-associated proteins; (**E**) Claudin-1 protein; (**F**) Occludin protein; (**G**) ZO-1 protein; (**H**) Immunofluorescence staining showing the distribution and expression of TJ-associated proteins in Caco-2 cells treated with 6-shogaol and/or PA (magnification 200×). Claudin-1, occludin, and ZO-1 proteins (green) were labeled with fluorescent secondary antibodies, and the nuclei (blue) were labeled with 4′,6-diamidino-2-phenylindole (DAPI); (**I**) Quantification of the Claudin-1 fluorescence intensity; (**J**) Quantification of the Occludin fluorescence intensity; (**K**) Quantification of the ZO-1 fluorescence intensity. Data are derived from three independent experiments and given as means ± standard deviations (n = 3). * *p* < 0.05, ** *p* < 0.01, *** *p* < 0.001 vs. the control; ^#^ *p* < 0.05, ^##^ *p* < 0.01, ^###^ *p* < 0.001 vs. the PA-treated group.

Figure 3 illustrates the effects of 6-shogaol on the mRNA and protein expressions of TJ-associated proteins (i.e., claudin-1, occludin, ZO-1) in Caco-2 cells with or without the PA treatment. Compared with the control, the PA treatment significantly (*p* < 0.01) decreased the mRNA expression levels of the three TJ-associated proteins (approximately 62%, 49%, and 36%, respectively, for claudin-1, occludin, and ZO-1) (Figure 3A–C). The presence of 6-shogaol significantly counteracted these PA-induced changes in a concentration-dependent fashion. Western blot analysis confirmed the effects of 6-shogaol on the expression of these TJ-associated proteins in Caco-2 cells (Figure 3D). Consistent with the qRT-PCR results, the 6-shogaol treatment significantly (*p* < 0.05) alleviated the PA-induced decreases in the protein expression of the three TJ-associated proteins (Figure 3E–G). Among the 6-shogaol treatments, 6-shogaol at 2.5 μM showed the greatest protective effect against PA-induced TJ damage in Caco-2 cells. This dose was selected for subsequent experiments to explore the underlying mechanisms. Compared with the control, the treatment with 6-shogaol at 1.25 or 2.5 μM in the absence of PA significantly upregulated the protein expression levels of these TJ proteins in Caco-2 cells. Immunofluorescence staining allowed visualization of the distribution and expression of these TJ-associated proteins in Caco-2 cells: continuous belt-like fluorescence (labeling of claudin-1, occludin, and ZO-1 proteins) was found around the borders of Caco-2 cells for the control group and the 6-shogaol-treated group (Figure 3H). The exposure to PA led to patchy/discontinuous immunostaining and significantly decreased the intensity of the fluorescence from the stained TJ-associated proteins. In comparison, the co-treatment with PA and 6-shogaol partially attenuated these PA-induced changes (Figure 3H–K). Taken together, 6-shogaol could protect Caco-2 cells against PA-induced damage to TJs.

### 3.4. 6-Shogaol Mitigated PA-Induced Inflammation in Caco-2 Cells

**Figure 4:**
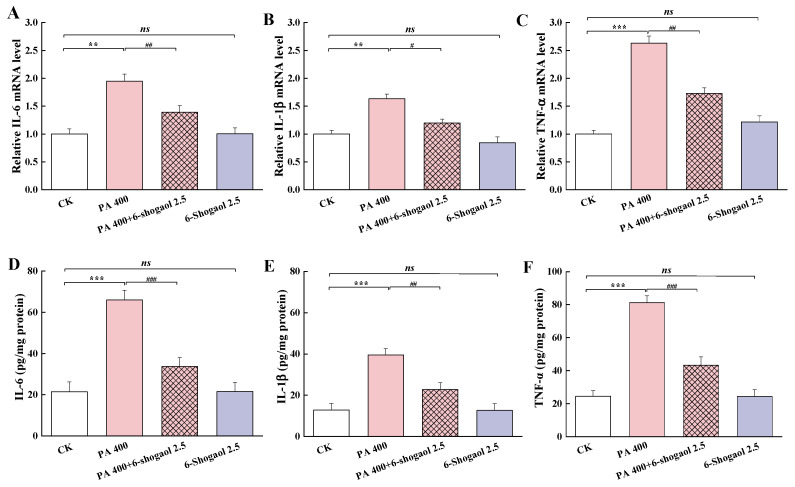
6-Shogaol mitigated PA-induced production of pro-inflammatory cytokines in Caco-2 cells: (**A**) Interleukin-6 (IL-6) mRNA; (**B**) IL-1β mRNA; (**C**) Tumor necrosis factor alpha (TNF-α) mRNA; (**D**) IL-6 level; (**E**) IL-1β level; (**F**) TNF-α level. Data are given as means ± standard deviations (n = 3). ** *p* < 0.01, *** *p* < 0.001 vs. the control; ^#^ *p* < 0.05, ^##^ *p* < 0.01, ^###^ *p* < 0.001 vs. the PA-treated group; *ns* indicates no significant difference between the 6-shogaol-treated group and the control at *p* < 0.05.

Figure 4 shows the effects of 6-shogaol on the expressions of the pro-inflammatory cytokines in Caco-2 cells with and without exposure to PA. Compared with the control, the PA treatment significantly (*p* < 0.01) increased the mRNA expression levels of three pro-inflammatory cytokines, IL-6, IL-1β, and TNF-α (Figure 4A–C), while the presence of 6-shogaol significantly counteracted these PA-induced changes. The PA alone treatment led to remarkably (*p* < 0.001) higher levels of these pro-inflammatory cytokines (increased by 208.1%, 209.5%, and 232.3% for IL-6, IL-1β, and TNF-α, respectively, compared with the control). The 6-shogaol treatment significantly (*p* < 0.01) counteracted such PA-induced increases, with the levels of IL-6, IL-1β, and TNF-α being suppressed by 48.9%, 42.4%, and 46.8%, respectively, in comparison with the group treated by PA alone. There were no significant differences in these inflammation-related markers between the 6-shogaol alone group and the control group.

### 3.5. 6-Shogaol Inhibited PA-Induced Activation of the TLR4/NF-κB Signaling Pathway

**Figure 5:**
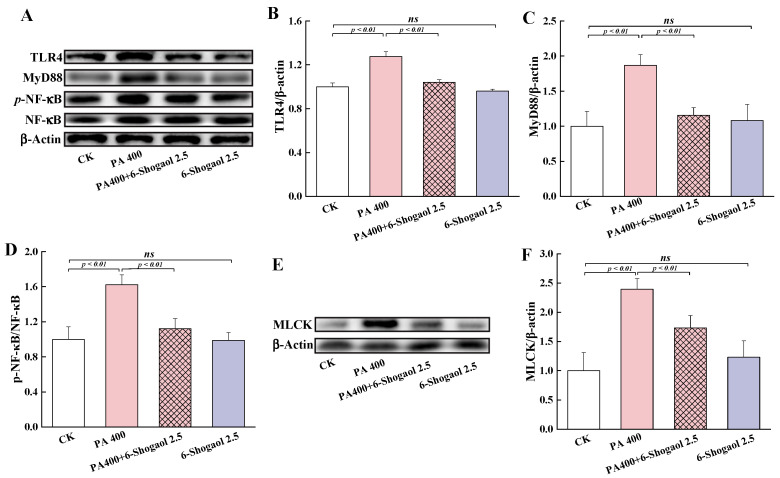
Effects of 6-shogaol on the TLR4/NF-κB signaling pathway and the expression of MLCK protein in Caco-2 cells treated with PA: (**A**) Representative immunoblotting of TLR4, MyD88, p-NF-κB, and NF-κB; (**B**) Relative expression of TLR4; (**C**) Relative expression of MyD88; (**D**) The *p*-NF-κB/NF-κB ratio; € Representative immunoblotting of MLCK; (**F**) Relative expression of MLCk. β-Actin was used as the loading control for the quantification of TLR4, MyD88, and MLCK. Data are given as means ± standard deviations (n = 3). *ns* indicates no significant difference between the 6-shogaol-treated group and the control at *p* < 0.05.

The TLR4/NF-κB signaling pathway is known for its vital role in regulating inflammatory responses. Therefore, it is important to examine whether the protective effect of 6-shogaol against PA-induced TJ damage was associated with this pathway. Figure 5A shows the representative immunoblots of the TLR4, MyD88, NF-κB, and *p*-NF-κB proteins in the different groups of Caco-2 cells. The exposure of cells to PA significantly (*p* < 0.01) increased the levels of TLR4 and MyD88 and the phosphorylation of NF-κB (p65) (as indicated by the *p*-NF-κB/NF-κB ratio) compared with the control (Figure 5B–D). The co-treatment with PA and 6-shogaol significantly (*p* < 0.01) suppressed these PA-induced changes. There were no significant differences in these inflammation-related regulatory proteins between the 6-shogaol alone group and the control group. Moreover, whether the 6-shogaol treatment could affect intestinal-barrier-related MLCK protein was also investigated in this study. As shown in Figure 5E,F, the expression of the MLCK protein was significantly (*p* < 0.01) upregulated in the PA alone group compared with the control but was significantly (*p* < 0.01) downregulated in the group co-treated with PA and 6-shogaol compared with PA alone.

### 3.6. MiRNA-216a-5p Mediated the Protective Effect of 6-Shogaol against TJ Damage in Caco-2 Cells

#### 3.6.1. MiRNA-216a-5p Directly Targeted TLR4

**Figure 6:**
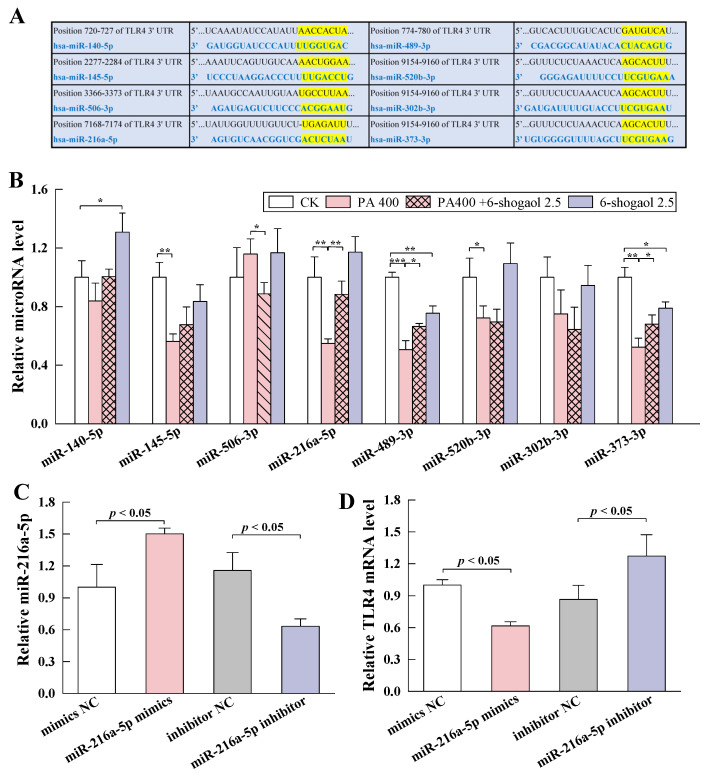
MiRNA-216a-5p directly targeted TLR4 mRNA: (**A**) The predicted TLR4-targeting miRNAs and their binding sites to the 3′-untranslated region (3′ UTR) of TLR4; (**B**) Relative levels of the predicted miRNAs in Caco-2 cells treated with 6-shogaol in the presence and absence of PA; (**C**) Relative expression of miR-216a-5p in Caco-2 cells transfected with miR-216a-5p mimics or inhibitor; (**D**) Effects of miR-216a-5p on the level of TLR4 mRNA. Data are given as means ± standard deviations (n = 3). * *p* < 0.05, ** *p* < 0.01, *** *p* < 0.001.

Multiple lines of evidence indicate the important roles of miRNAs as regulators of inflammatory responses and other pathological processes [22]. To further explore the molecular mechanism by which 6-shogaol protected Caco-2 cells against PA-induced TJ damage, the miRNAs that possibly interacted with TLR4 mRNA were screened based on miRNA target gene prediction databases (TargetScan, miRDB, and PicTar). As shown in Figure 6A, eight miRNAs were identified as having potential to target the 3′-untranslated region (3′UTR) of TLR4: miR-140-5p, miR-145-5p, miR-506-3p, miR-216a-5p, miR-489-3p, miR-520b-3p, miR-302b-3p, and miR-373-3p. The qRT-PCR experiments (Figure 6B) showed that compared with the control, the PA treatment significantly downregulated the expression of miR-145-5p (*p* < 0.01), miR-216a-5p (*p* < 0.01), miR-489-3p (*p* < 0.001), miR-520b-3p (*p* < 0.05), and miR-373-3p (*p* < 0.01). Among these miRNAs, the expression of miR-216a-5p, miR-489-3p, and miR-373-3p was significantly upregulated following the co-treatment with PA and 6-shogaol, with a significantly greater increase (62.2%) for miR-216a-5p than for the other miRNAs. There was no significant change for miR-216a-5p between the 6-shogaol alone group and the control. These findings are in agreement with the predicted results shown in Figure 6A: other miRNAs were not effective or less effective than miRNA-216a-5p in directly targeting the TLR4 mRNA. Therefore, miRNA-216a-5p was selected in subsequent experiments.

As shown in Figure 6C, miRNA-216a-5p was overexpressed when the cells were transfected with miRNA-216a-5p mimics, whereas the expression of miRNA-216a-5p was inhibited when the cells were transfected with an miRNA-216a-5p inhibitor, indicating that miRNA-216a-5p was successfully transfected into Caco-2 cells. Figure 6D shows the effects of miRNA-216a-5p on the expression of TLR4 mRNA. While the miRNA-216a-5p mimics led to a significant (*p* < 0.05) decrease in the mRNA level of TLR4, the miRNA-216a-5p inhibitor exerted the opposite effects. These results are in agreement with a previous finding that overexpression of miRNA-216a-5p could significantly reduce the luciferase activity of wild-type TLR4 3′ UTR plasmid [18]. Taken together, the miRNA-216a-5p/TLR4 axis may mediate the protective effect of 6-shogaol against PA-induced TJ damage.

#### 3.6.2. MiRNA-216a-5p Was Involved in the Protection Provided by 6-Shogaol against PA-Induced TJ Damage

**Figure 7:**
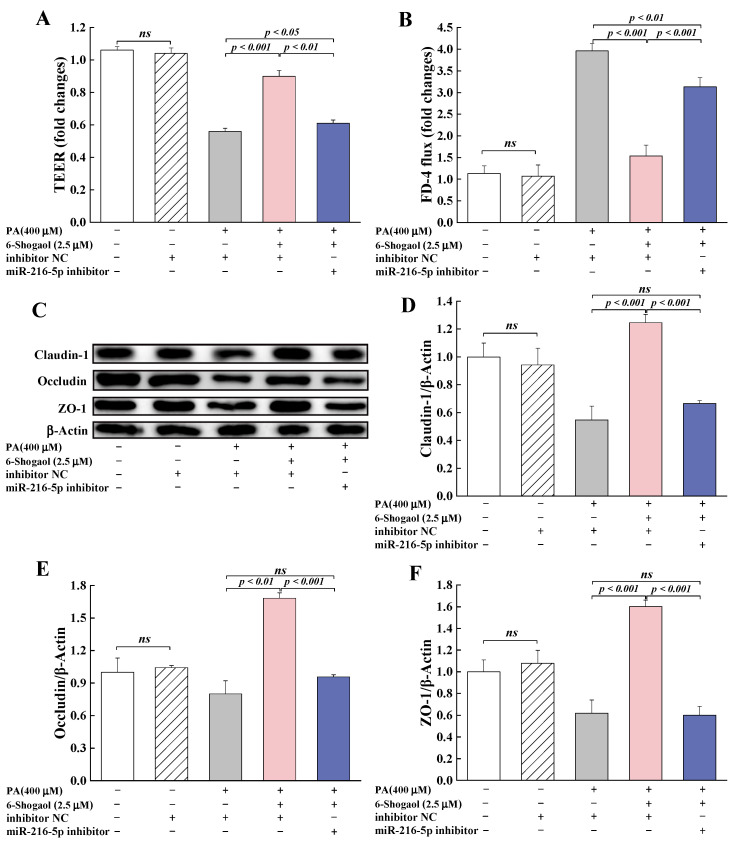
MiRNA-216a-5p mediated the protection provided by 6-shogaol against PA-induced TJ disruption: (**A**) Effect of miRNA-216a-5p on TEER in Caco-2 monolayers; (**B**) Effect of miRNA-216a-5p on FD-4 flux in Caco-2 monolayers; (**C**) Representative immunoblotting of TJ-associated proteins in Caco-2 cells transfected with miRNA-216a-5p inhibitor; (**D**–**F**) Effects of miRNA-216a-5p on the expression of claudin-1, occludin, and ZO-1 proteins in Caco-2 cells. Data are given as means ± standard deviations (n = 3). *ns* indicates no significant difference at *p* < 0.05.

**Figure 8:**
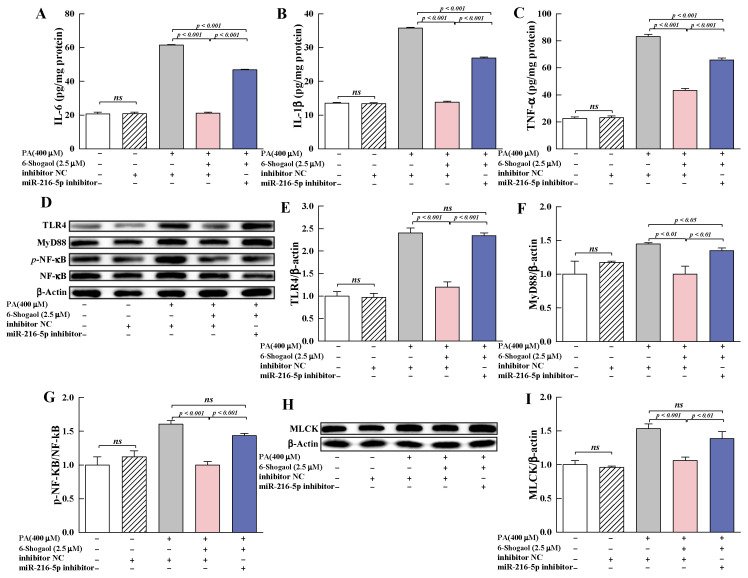
Effects of miR-216a-5p on pro-inflammatory cytokines and the TLR4/NF-κB signaling pathway: (**A**–**C**) Effects of miR-216a-5p on the levels of IL-6, IL-1β, and TNF-α; (**D**) Representative immunoblotting of TLR4, MyD88, p-NF-κB, and NF-κB; (**E**–**G**) Effects of miR-216a-5p on the expression of TLR4 and MyD88, and the phosphorylation of NF-κB; (**H**) Representative immunoblotting of MLCK; (**I**) Effects of miR-216a-5p on the expression of MLCK. Data are given as means ± standard deviations (n = 3). *ns* indicates no significant difference at *p* < 0.05.

To further investigate the roles of miR-216a-5p in the protective effect of 6-shogaol against PA-induced TJ damage, miR-216a-5p inhibitor was transfected into Caco-2 cells, and the changes of TEER, FD-4 flux and TJs were determined. Compared with the group co-treated with 6-shogaol and PA, miR-216a-5p inhibitor significantly (*p* < 0.001) decreased TEER; increased FD-4 flux; and suppressed the expression of claudin-1, occludin, and ZO-1 proteins in the cells (Figure 7). Furthermore, miR-216a-5p inhibitor significantly (*p* < 0.001) suppressed the protective effect of 6-shogaol against PA-induced inflammation in Caco-2 cells, as evidenced by the lower levels of IL-6, IL-1β, and TNF-α for the PA+6-shogaol+miR-216a-5p inhibitor group than for the PA+6-shogaol group (Figure 8A–C). The presence of MiR-216a-5p inhibitor led to significant upregulation of the expression of TLR4 and MyD88 and the phosphorylation of NF-κB and MLCK in Caco-2 cells (Figure 8D–I). It is worth noting that the effects of miR-216a-5p inhibitor on these indices were similar to those of the PA treatment. Taken together, this research reveals for the first time that the miR-216a-5p/TLR4/NF-κB signaling pathway is involved in the protective effects of 6-shogaol against PA-induced TJ damage in Caco-2 cells.

## 4. Discussion

The intestinal epithelium, consisting of a single layer of various cells, plays a crucial role in maintaining the host’s homeostasis and health. The intestinal epithelium acts as a physical barrier/fence against diverse luminal noxious agents (e.g., pathogenic microorganisms, toxins, and allergens), a controlled repository and homeostatic system for molecular transit, an integral part of innate immunity, and a central coordinator for crosstalk between immune cells and the microbiota [23,24]. TJs are composed of transmembrane proteins (e.g., claudin and occludin) connected to the actin cytoskeleton through a cytoplasmic scaffolding protein network (e.g., ZO-1, -2, and -3). TJs form a controllable intercellular barrier and act as multifunctional and selective gates for the paracellular permeability of the intestinal epithelium [25]. TJs are also responsible for the integration and transmission of signals and involved in the regulation of the gene expression required for cell proliferation and differentiation [26]. TJ dysfunction and derived paracellular hyperpermeability take place in a number of clinical conditions, especially autoimmune and immune-mediated disorders such as inflammatory bowel diseases (IBDs), celiac diseases, food allergies, irritable bowel syndrome (IBS), diabetes, and obesity [3].

High-fat and/or high-sugar diets are known to have adverse effects on human health by inducing gut microbiota dysbiosis, triggering/exacerbating intestinal barrier dysfunction, and initiating/promoting metabolic disorders and the inflammatory response [27]. For example, HFD feeding for 4 weeks was reported to greatly increase the intestinal permeability and reduce the mRNA levels of ZO-1 and occludin in mice [28]. HFD feeding for 11 and 22 weeks disrupted the mucosal barrier integrity and decreased the expression of claudin-1 in mice [29]. PA, as one of the most common long-chain saturated fatty acids in human diets, has lipotoxic effects on the different organs of humans [30]. In this study, we found that PA at 400 μM or below did not induce a detectable toxic insult to the Caco-2 monolayers. Such a result is in agreement with previous reports [31]. These PA concentrations are within the physiological postprandial intestinal concentration range of free fatty acids (FFAs) in human plasma (i.e., 0.2~2 mM). Furthermore, deleterious effects of PA on the intestinal permeability were found. In this study, a 24-h PA treatment was found to increase the paracellular permeability of Caco-2 monolayers, as evidenced by a 48% reduction in the TEER values and 4.1-fold increase in the FD-4 flux compared with the control. PA-induced TJ barrier disruption was further confirmed by qRT-PCR, Western blotting, and immunofluorescence assays (significant decreases in the mRNA and protein expression of TJ-associated proteins, i.e., claudin-1, occludin, and ZO-1, in the cells treated with PA). These results suggest that a PA-induced intestinal barrier dysfunction model was successfully established. Li et al. (2021) reported that the treatment with 50 μM PA led to increased permeability of the blood–brain barrier (BBB) and reduced expression of ZO-1 and occludin proteins in murine brain endothelial bEnd.3 cells [7]. Gori et al. (2020) compared the effects of PA, bacterial LPS, and ethanol on the integrity of the gut epithelium using a Caco-2 cell model. They found that all these substances significantly increased the FD-4 permeability across the Caco-2 cell monolayer, but the effect exerted by PA appeared to be faster and more significant than those of LPS and ethanol [31]. Ghezzal et al. (2020) showed that unlike unsaturated oleic acid, a 24 h-treatment with PA at a dose of 600 μM could significantly decrease the expression of junction proteins (ZO-1, occludin, tricellulin, and E-cadherin) in Caco-2/TC7 cells without altering their localizations at cell–cell boundaries and during cell–cell contacts [10].

In this study, PA was also found to increase the production of pro-inflammatory cytokines, IL-6, IL-1β, and TNF-α, suggesting increased intestinal permeability accompanied by the inflammatory response in the intestinal epithelium. These results agree with previous findings that PA was able to modulate the immune system by inducing monocyte activation and stimulating the pro-inflammatory response in human immune cells [32]. The pro-inflammatory cytokine-mediated dysfunction of the TJ barrier is an important contributor to the initiation and/or development of several intestinal and systemic diseases [26]. TNF-α is mainly produced by activated T cells and macrophages and can induce apoptosis and the inflammatory response in intestinal epithelial cells [2]. TNF-α-treated Caco-2 cells exhibit reduced TEER, increased inulin permeability, decreased expression of ZO-1 proteins, and elevated MLCK and myosin light-chain (MLC) phosphorylation [33]. An increase in IL-1β in the intestinal mucosa contributes to the increased permeability of intestinal TJs by regulating the expression and redistribution of occludin and the phosphorylation of MLC [34]. IL-6 is a pleiotropic cytokine responsible for the host immune response to various infections and is mainly produced by the intestinal epithelial cells and lamina propria mononuclear cells in IBD [35]. IL-6 was found to induce Claudin-2 expression at a transcriptional level and in a Cdx2-dependent fashion in the intestinal Caco-2 cells [36]. The presence of IL-6 decreased the protein expression of ZO-1 and Occludin and increased intestinal permeability, causing intestinal barrier dysfunction in mice resuscitated from hemorrhagic shock [37].

The importance of dietary therapeutics in maintaining intestinal health is well recognized. The positive roles of certain nutrients and phytochemicals in regulating the integrity and functions of the intestinal barrier and treating inflammatory diseases have been studied and reported, though the underlying mechanisms are not yet fully clear [2]. In this study, the PA-induced abnormalities in the biomarkers associated with the intestinal barrier functions of Caco-2 cells were significantly suppressed upon treatment with 6-shogaol (i.e., PA (400 μM) + 6-shogaol (2.5 μM) for 24 h). These results suggest that 6-shogaol might be a promising agent for treating PA-induced intestinal barrier dysfunction. Interestingly, we found that even in the absence of PA, 6-shogaol possessed the ability to enhance the intestinal barrier function of Caco-2 cells (i.e., a higher TEER value, lower FD-4 flux, and increased expression of TJ-associated proteins compared with the control). Similar effects were also observed in previous studies on other phenolic compounds (i.e., quercetin and kaempferol) using Caco-2 cells [38,39].

Pro-inflammatory cytokines are positive mediators of inflammation and key biomarkers of immunomodulation and thus have been used as potential therapeutic targets. Considering the crucial roles of pro-inflammatory cytokines in PA-induced intestinal barrier dysfunction, the effects of 6-shogaol on the levels of pro-inflammatory cytokines (IL-6, IL-1β, and TNF-α) were also examined in this study. 6-Shogaol attenuated the PA-initiated increases in the levels of these pro-inflammatory cytokines, suggesting the anti-inflammatory effects of 6-shogaol on the PA-induced immune response. Previous studies revealed that 6-shogaol ameliorated diabetic nephropathy in db/db mice partly through its anti-inflammatory action by decreasing the TNF-α, monocyte chemotactic protein-1 (MCP-1), and IL-6 levels in the circulation and kidneys while suppressing NF-κB expression [40]. 6-Shogaol could significantly suppress LPS-induced microglial activation in primary cortical neuron-glia culture and in an in vivo neuroinflammatory model by inhibiting the production of prostaglandin E2 and pro-inflammatory cytokines [41]. 6-Shogaol was found to be able to inhibit inflammation-related cell functions in LPS-activated human umbilical vein endothelial cells (HUVECs) [42].

As an important part of innate immunity, the TLR family plays a crucial role in identifying both endogenous and exogenous stimuli to trigger immune and inflammatory responses [43]. Long-term activation of TLR4 has been associated with autoimmune-related diseases, neurodegeneration, and cancers [44]. Once activated by various stimuli, TLR4 dimerizes and recruits MyD88. The interaction of MyD88 with the TIR domain of TLR4 leads to the recruitment of interleukin-1 receptor-associated kinase (IRAK)-4 and the phosphorylation of IRAK-1 induced by the phosphorylation of IRAK-4. The phosphorylation of IRAK-1 can trigger the degradation of IRAK-1, which is closely associated with TNF receptor-associated factor 6 (TRAF6), causing the activation of inhibitor κB kinase (IKK) complex and subsequent activation of the NF-κB signaling pathway [45]. As a result, excessive pro-inflammatory cytokines and chemokines are produced to induce inflammation. LPS has been identified as a natural TLR4 agonist, as LPS can bind to the hydrophobic pocket of MD-2 then inhibit the function of TLR4 [46]. PA acts as a TLR4 ligand and activates the NF-κB signaling pathway, leading to the secretion of IL-1β in human dendritic cells [32]. In this study, such a notion was further confirmed: the exposure to PA led to significant increases in the expression of TLR4 and MyD88 proteins and the phosphorylation of NF-κB; 6-Shogaol significantly suppressed such PA-induced increases, making the levels of these indicators comparable to those of the control group. Such results are in agreement with previous findings that 6-shogaol was able to suppress LPS-induced dimerization of TLR4 and the activation of the NF-κB pathway in RAW264.7 macrophages [45]. MLCK plays a vital role in the assembly and regulation of TJs via the phosphorylation of MLC. It is known that the activated NF-κB translocates to the nucleus and binds to the *cis*-κB binding site on the MLCK promoter region, causing MLCK-mediated MLC phosphorylation concomitant remodeling of TJ proteins (e.g., actin cytoskeleton) and localization of TJ proteins by contracting actin-myosin filaments, and eventually resulting in an impaired intestinal barrier [47]. In this study, along with the activation of the NF-κB pathway, the PA treatment significantly promoted the expression of MLCK in Caco-2 cells. This finding was also obtained previously in an LPS-induced colonic TJ disruption model of rats [48] and in TNF-α-treated Caco-2 cells [49]. In this study, 6-shogaol suppressed PA-induced increases in the expression of MLCK protein, which was associated with the inhibitory effects of 6-shogaol on the NF-κB signaling pathway. Collectively, these results suggest that 6-shogaol protected Caco-2 cells against PA-induced TJ dysfunction and intestinal barrier impairment by inhibiting the activation of the TLR4/MyD88/NF-κB signaling pathway and the expression of MLCK protein.

**Figure 9:**
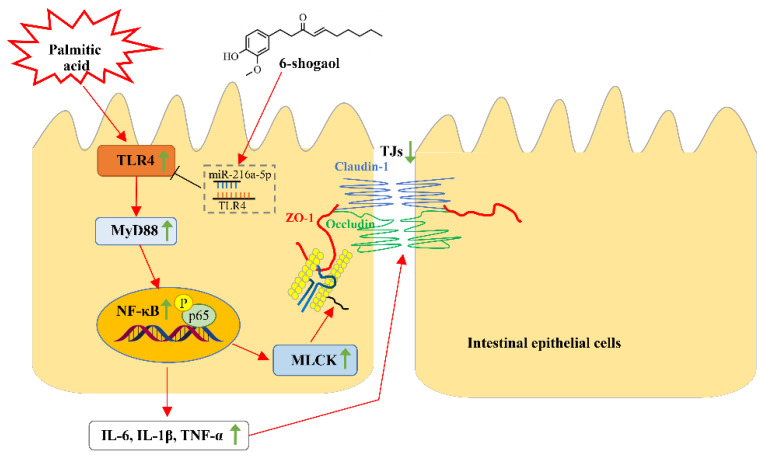
A schematic diagram illustrating the possible mechanisms underlying the protective effects of 6-shogaol against PA-induced intestinal barrier dysfunction. Green arrows ↑ and ↓ indicate that the proteins or cytokines are upregulated and downregulated, respectively.

It is well established that miRNAs can post-transcriptionally regulate TJs and modulate the epithelial and endothelial barrier functions in different tissues, including the intestinal epithelium [50]. For example, miR-21 was found to be able to prevent intestinal barrier dysfunction by increasing the expression of occludin via targeting of Rho-associated protein kinase 1 [51]. MiR-93 was reported to be capable of affecting claudin-3 expression by regulating protein tyrosine kinase 6 and improving the intestinal epithelial dysfunction during an inflammatory injury [52]. In this study, we screened out the TLR4-targeted miRNAs from multiple databases (TargetScan, miRDB, and PicTar) to further elucidate the molecular mechanism(s) by which 6-shogaol alleviated PA-induced intestinal barrier impairment. In this research, the PA alone treatment decreased the level of miR-216a-5p whilst the presence of 6-shogaol together with the PA treatment significantly lessened the PA-induced decrease in miR-216a-5p. Therefore, it is speculated that miR-216a-5p may be involved in the protective action of 6-shogaol against PA-induced intestinal barrier impairment and the inflammatory response by targeting TLR4. This research also confirmed that inhibition of miRNA-216a-5p was able to significantly counteract PA-induced TJ dysfunction, which was closely associated with the regulatory effects of miRNA-216a-5p on the TLR4/MyD88/NF-κB pathway and MLCK proteins. Figure 9 shows the possible mechanisms underlying the protective effects of 6-shogaol against PA-induced TJ dysfunction and intestinal barrier impairment.

## 5. Conclusions

This research demonstrated for the first time that 6-shogaol can attenuate PA-induced intestinal barrier dysfunction in Caco-2 cells by regulating TJ-associated proteins via the miR-216a-5p/TLR4/NF-κB axis and modulating the expression of MLCK. These findings suggest that 6-shogaol might be a promising food-derived agent for treating PA-induced intestinal epithelial barrier impairment and derived intestinal disorders. Further studies are needed to elucidate the protective effects of 6-shogaol against PA-induced TJ dysfunction in animal models and the molecular mechanism(s) by which 6-shogaol modulates miR-216a-5p.

## Figures and Tables

**Table 1:** The sequences of primers used in the qRT-PCR assays.

Genes	Primer	Sequence
Claudin-1	Forward	CACCGTCTGTGTTTGAGCA
	Reverse	CAAACCACCGCTTACAGATG
Occludin	Forward	GACTATGTGGAAAGAGTTGAC
	Reverse	ACCGCTGCTGTAACGAG
ZO-1	Forward	TTCACGCAGTTACGAGCAAG
	Reverse	TTGGTGTTTGAAGGCAGAGC
IL-6	Forward	ACTCACCTCTTCAGAACGAATTG
	Reverse	CCATCTTTGGAAGGTTCAGGTTG
IL-1β	Forward	AGCTACGAATCTCCGACCAC
	Reverse	CGTTATCCCATGTGTCGAAGAA
TNF-α	Forward	GGCAGTCAGATCATCTTCTCGAA
	Reverse	TGAAGAGGACCTGGGAGTAGATG
GAPDH	Forward	CTCCTCCTGTTCGACAGTCA
	Reverse	CGACCAAATCCGTTGACTCC
U6	Forward	GGAACGATACAGAGAAGATTAGC
	Reverse	TGGAACGCTTCACGAATTTGCG
MiR-140-5p	Forward	GCGCAGTGGTTTTACCCTATGGTAG
MiR-145-5p	Forward	GTCCAGTTTTCCCAGGAATCCCT
MiR-506-3p	Forward	CGCTAAGGCACCCTTCTGAGTAGA
MiR-216a-5p	Forward	CGTAATCTCAGCTGGCAACTGTGA
MiR-489-3p	Forward	CCGTGACATCACATATACGGCAGC
MiR-520b-3p	Forward	CCGCAAAGTGCTTCCTTTTAGAGGG
MiR-302b-3p	Forward	CGCCGTAAGTGCTTCCATGTTTTAGT
MiR-373-3p	Forward	GGAAGTGCTTCGATTTTGGGGTGT

## Data Availability

Data is contained within this article.
